# Mitochondrial Damage in Myocardial Ischemia/Reperfusion Injury and Application of Natural Plant Products

**DOI:** 10.1155/2022/8726564

**Published:** 2022-05-16

**Authors:** Xin Su, Mingyang Zhou, Yingjian Li, Na An, Fan Yang, Guoxia Zhang, Lianjiang Xu, Hengwen Chen, Hongjin Wu, Yanwei Xing

**Affiliations:** ^1^Guang'anmen Hospital, China Academy of Chinese Medical Sciences, Beijing, China; ^2^Beijing Anzhen Hospital, Capital Medical University, Beijing, China; ^3^Beijing University of Chinese Medicine, Beijing, China; ^4^Hebei Gangkou Hospital, Qinhuangdao, Hebei, China; ^5^Boao International Hospital, Shanghai University of Traditional Chinese Medicine, Qionghai, China

## Abstract

Ischemic heart disease (IHD) is currently one of the leading causes of death among cardiovascular diseases worldwide. In addition, blood reflow and reperfusion paradoxically also lead to further death of cardiomyocytes and increase the infarct size. Multiple evidences indicated that mitochondrial function and structural disorders were the basic driving force of IHD. We summed up the latest evidence of the basic associations and underlying mechanisms of mitochondrial damage in the event of ischemia/reperfusion (I/R) injury. This review then reviewed natural plant products (NPPs) which have been demonstrated to mitochondria-targeted therapeutic effects during I/R injury and the potential pathways involved. We realized that NPPs mainly maintained the integrality of mitochondria membrane and ameliorated dysfunction, such as improving abnormal mitochondrial calcium handling and inhibiting oxidative stress, so as to protect cardiomyocytes during I/R injury. This information will improve our knowledge of mitochondrial biology and I/R-induced injury's pathogenesis and exhibit that NPPs hold promise for translation into potential therapies that target mitochondria.

## 1. Introduction

By the report of the World Health Organization in 2017, ischemic heart disease (IHD) accounts for 17.7 million deaths per year worldwide [1]. IHD remains one of the leading causes of death among cardiovascular diseases, which brings huge social and economic burden [2, 3]. Clinically, IHD principally includes coronary heart disease (angina, nonfatal myocardial infarction, and coronary death), asymptomatic myocardial ischemia, sudden cardiac death, and ischemic heart failure [4, 5]. Current treatment strategies for IHD mainly rely on pharmacological interventions such as statins, antiplatelet drugs, and beta-receptor blockers [6]. These drugs are used for condition stabilization and reduction of acute events. Alternatively, the blood supply can be restored immediately by surgical treatment [7, 8]. However, the reperfusion paradoxically causes further myocardial contractile dysfunction and cardiomyocyte death, a phenomenon known as ischemia/reperfusion (I/R) injury. The final outcome of I/R on damaged cardiomyocytes is myocardial infarction [9]. Myocardial repair after ischemia involves a series of intricate and fine inflammatory reactions [10]. Chronic remodeling occurs when the inflammatory response is unbalanced, eventually resulting in heart failure [11–13]. Protecting the heart from pathological reparative process is critical, and meaningful therapeutic strategies need to be continuously explored.

Natural plant products (NPPs) are defined as a large family of chemical entities with a wide variety of bioactive ingredients that originate mainly from fruit and medicine plants [14]. It can be roughly divided into several categories: polyphenols (approximately classified into flavonoids and nonflavonoids), saponins, terpenoids, and alkaloids. Many NPPs have pharmacological or biological activities, which can not only be applied as agents for treating diseases but also a promethean fire for the development of potential new drugs. In recent decades, NPPs has broad application prospects in the fields of pharmaceutical chemistry, molecular biology, and medicine. Epidemiological researches have found that the prevention of cardiovascular disease is related to a diet rich in NPPs [15, 16]. Several studies reported that NPPs regulate the balance of calcium homeostasis [17], protect mitochondrial function [18, 19], and alleviate free radicals [20, 21] during myocardial ischemia. Therefore, NPPs play different degrees of anti-ischemic effects in IHD, and the effects of antioxidative stress, anti-inflammatory, and antiapoptotic are particularly prominent. Mitochondrial damage is actually linked to oxidative stress, inflammation, and apoptosis. It is crucial to study the therapeutic strategy of mitochondrial damage during myocardial I/R. This review will summarize the pathological mechanism of mitochondrial damage and the status and process of the protective effect of NPPs in IHD.

## 2. Mitochondrial Damage in Ischemia/Reperfusion Injury

Mitochondria are essential organelles that not merely perform energy metabolism and many biosynthetic but also contribute to stress responses such as apoptosis [22]. They form a dynamic, interrelated network with other organelles for normal mitochondrial and cellular functions. Mitochondrial membrane is a biofilm enclosing mitochondria, consisting of an inner and an outer unit membrane. The outer mitochondrial membrane (OMM) is similar in composition to other cellular components and contains a pore formed by the voltage-dependent anion channel (VDAC) that enables interchange between the mitochondria and the cytosol [23]. The area of mitochondrial inner membrane is greatly enhanced by folding internally into cristae, which is conducive to effective oxidative phosphorylation [24]. The core of mitochondrial energy metabolism is the tricarboxylic acid cycle, which uses pyruvate from glycolysis to produce acetyl-CoA and breaks down the acetyl moiety into carbon dioxide, with electrons entering nicotinamide adenine dinucleotide (NADH) or the coenzyme Q (CoQ) pool. From the CoQ pool, the reduction potential difference drives the movement of electrons through mitochondrial respiratory chain complex I, III, and IV, where electrons then reduce oxygen to water. This process establishes the mitochondrial membrane potential (MMP) of ~150-160 mV [25]. In addition, mitochondrial adenosine triphosphate (ATP) synthesis can drive ATP-dependent work in the cytoplasm [26]. Mitochondria are dynamic organelles whose morphology maintains a balance between fusion and fission events. There are many kinds of related regulatory proteins, such as mitofusin and optic atrophy protein 1 (OPA1) [27–29], and mitophagy is also involved. The function of mitochondria extends beyond the boundaries of organelles and is particularly important in the response of cells or tissues to stress. So be not at all surprising that mitochondrial dysfunction has emerged as a key factor in a variety of cardiovascular diseases, including I/R injury.

**Figure 1:**
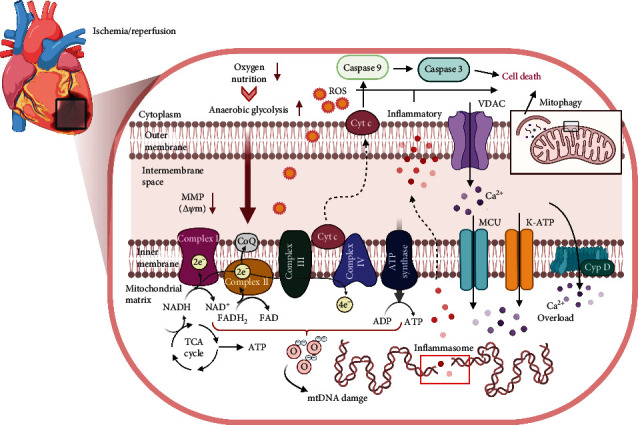
Mitochondrial damage in ischemia/reperfusion injury. Cyt c: cytochrome c; TCA: tricarboxylic acid cycle; NADH: nicotinamide adenine dinucleotide; ROS: reactive oxygen species; MMP: mitochondrial membrane potential; ADP: adenosine diphosphate; FADH: flavin adenine dinucleotide; ATP: adenosine triphosphate; mtDNA: mitochondrial DNA; VDAC: voltage-dependent anion channel; MCU: mitochondrial Ca^2+^ uniporter; K-ATP: adenosine triphosphate-sensitive potassium channel; Cyp D: cyclophilin D. Created with http://BioRender.com.

Mitochondria electron transport sustains progressive injury during cardiac ischemia [30]; a decline in mitochondrial energy metabolism is noted. Although energy metabolism can be restored during reperfusion, the early mitochondrial calcium overload and reactive oxygen species (ROS) increase can promote the opening of mitochondrial permeability transition pore (mPTP) and lead to the collapse of MMP, eventually causing cell death [31]. In summary, the pathologic mechanism regarding how mitochondria are involved in I/R injury has emerged. Important among these are ROS overproduction, impaired electron transport chain activity, calcium dyshomeostasis, aberrant mPTP opening, MMP depolarization, and inappropriate activation of apoptosis (Figure 1). In the following sections, we will discuss these mechanisms in greater depth and clarify the potential of reducing I/R injury via mitochondrial-related pathways.

### 2.1. Oxidative Stress

Limited oxygen, energy consumption, and ion homeostasis changes produce ROS in myocardial cells during the ischemic period [32]. Ischemic injury occurs in a progressive, time-dependent manner. During prolonged ischemia (>60 min), all components of the respiratory chain may be damaged by oxidative stress [33]. During reperfusion, a reperfusion oxidant burst can cause myocardial cell death. The low ratio of NADH to nicotinamide adenine dinucleotide^+^ (NAD^+^) ensures sufficient redox driving force to maintain adequate MMP for ATP synthesis under physiological conditions. When low flow ischemia and hypoxia are applied, complex I increases the ratio of NADH: NAD^+^ and reduces the CoQ pool. Complex I is responsible for the accumulation of mitochondrial succinate via complex II and the loss of MMP, thereby halting ATP synthesis [34]. When reintroduced O_2_ encounters a reduced CoQ pool during reperfusion, complexes III and IV further reduce CoQ pool and enhance the reverse effect under ischemia period. Finally, O_2_ is converted to O_2_^•−^ via the prosthetic group of complex I, producing a burst of ROS that may lead to myocardial cell death [35]. Besides, succinate dehydrogenase (SDH), as the complex II of the mitochondrial respiratory chain, transfers the electrons of succinate to the respiratory chain, thereby provoking electron transport chain by oxidizing succinate [36]. Within a few minutes after reperfusion, the accumulated succinate can be rapidly oxidized by SDH, which promotes the generation of extensive ROS by reverse electron transfer through complex I in mitochondria [37, 38]. The opening of mPTP in I/R injury enhances the ROS generation. Superoxide increases after mPTP opening and is at a much greater rate in the presence of succinate. This may be the result of mitochondrial loss of cytochrome c (Cyt c), which is important for scavenging ROS [39]. Increased superoxide production due to mPTP opening may lead to further opening of mPTP in adjacent mitochondria [40].

In addition, the activation of prosurvival kinases, such as phosphoinositide-3-kinase (PI3K)/threonine-protein kinase (Akt) and extracellular signal-regulated kinase 1/2 (ERK1/2) (collectively known as “reperfusion injury salvage kinase pathway”), has been demonstrated to exert cardioprotection against I/R injury [41]. These signaling pathways seem to focus on mitochondria, especially targeting mPTP in response to oxidative stress and ATP depletion [42, 43]. Shanmugam et al. [44] used two myocardial I/R models: ligation of the left anterior descending artery and Langendorff-isolated heart perfusion system. The group confirmed that the activation of PI3K/Akt pathway in the rat heart reduced the level of myocardial oxidative stress and preserved mitochondrial function. Other research findings [45, 46] speculate that the G protein-coupled estrogen receptor 1 and paraoxonase 2, as upstream moleculars, activate reperfusion injury salvage kinase pathway and play an active role in protecting myocardial mitochondria and inhibiting oxidative stress.

### 2.2. Mitophagy

Autophagy is a vital part of the intracellular cell homeostasis, working with the ubiquitin/proteasome system [47]. Targeted substrates are modified to be ubiquitinated and then recognized by ubiquitin receptors, which either translocate substrates to the proteasome or self-degradation by the phagosome. Autophagy is further divided into selective autophagy and nonselective autophagy. Selective autophagy is mediated by unique autophagy receptors and is classified according to the specific substrate being degraded. One of them is mitophagy, which specializes in identifying and degrading protein aggregates, waste lipids, and defective mitochondria. Mitophagy is important for the maintenance of mitochondrial quality control and can be regulated by a variety of conditions, such as cell differentiation, hypoxia, or lack of nutrients and ATP [48–50]. Recently, it has been proved that I/R injury can induce abnormal mitophagy resulting in dysfunctional mitochondria accumulation and cell death [51, 52].

The phosphatase and tensin homolog-induced putative kinase 1 (PINK1)/Parkin pathway is a canonical ubiquitin-mediated mitophagy pathway. PINK1, as a mitochondrial polarization sensor, enters mitochondrial intermembrane space through OMM translocase and binds to the inner membrane by into translocase of inner mitochondrial membrane in the polarized mitochondria, which can be hydrolyzed by a variety of proteasomes [53, 54]. The mechanism is therefore preserved at low levels within cardiomyocytes under physiological conditions. However, when mitochondrial is damaged by stress, such as I/R injury, MMP decreases and PINK1 accumulates in the OMM. PINK1 then phosphorylates and transfers Parkin from the cytoplasm to the OMM. Parkin ubiquitinates mitochondria so that mitochondria are recognized by autophagosomes. It is reported that overactivation of PINK1/Parkin-mediated mitophagy is harmful for myocardial cells. Ji et al. [55] confirmed that I/R induced by 30 minutes of ischemia followed by 120 minutes of reperfusion induced excessive PINK1/Parkin-mediated mitophagy in cardiomyocyte, thereby leading to death. FUN14 domain containing 1 (FUNDC1) plays an increasingly important role in mitophagy under hypoxic condition. The prevailing view is that FUNDC1-mediated mitophagy has been regarded as a protective property in myocardial I/R injury. The mitochondrial unfolded protein response involves in the activation of proteases, chaperones, and antioxidant enzymes and is used to degrade abnormal or unfolded proteins and restore mitochondrial function. FUNDC1 activates the mitochondrial unfolded protein response to inhibit mitochondrial oxidative stress and stimulate mitochondrial biogenesis under myocardial I/R injury [56]. In addition, OPA1 is predominately embedded in the mitochondrial membrane, intimately linked to the degradation of damaged mitochondria through mitophagy [57]. Guan et al. [58] discovered that the expression of mitochondrial calcium transporter increased during I/R condition, which was related to myocardial injury and mitophagy, and downregulated the expression of OPA1. After inhibiting calpain, OPA1 inhibited mitochondrial excessive division and apoptosis. OPA1 expression is potentially regulated by the adenosine 5′-monophosphate-activated protein kinase (AMPK) pathway, which is also found to be related with mitophagy modification [59–61].

### 2.3. Inflammatory

Myocardial I/R injury involves inflammatory cell recruitment, together with the activation of innate and adaptive immune reactions [62–64]. The excessive inflammation-related process contributes to adverse outcomes, such as metabolic dysfunction, oxidative stress response, degeneration, and necrosis. Mitochondria are increasingly being recognized as having critical roles in activating and moderating a battery of inflammation-related processes. Many products in mitochondria can trigger inflammation; ROS from mitochondrial respiratory chain can directly activate nucleotide-binding oligomerization domain-like receptor family pyrin domain containing 3 (NLRP3) [65]. NLRP3 inflammasome recruits and activates proinflammatory proteases that contribute to the release of mature cytokines [66]. NLRP3 inflammasome senses mitochondrial dysfunction. The NLRP3 inflammasome induces mitochondrial damage through mitochondrial ROS, and inhibition of the opening of mPTP could effectively inhibit the activation of NLRP3 inflammasome and ROS generation from mitochondria [67]. In addition, mitochondrial DNA, as an endogenous damage associated molecular pattern, has been shown to activate inflammasomes [68, 69]. Qin et al. [70] confirmed that DNA released from damaged mitochondria promoted the release of mature inflammatory factors, such as interleukin 6 and interleukin 8, and the resulting inflammatory damage contributed to myocardial I/R injury. Nakahira et al. [71] clarified that inflammation was caused by mitochondrial DNA released into the cytoplasm that activated the NLRP3 inflammasome. The opening of mPTP and inflammation cooperatively progress myocardial I/R injury. Ikeda et al. [72] investigated the synergistic effect of cyclophilin D (Cyp D, a key molecule for mPTP opening) and C-C chemokine receptor 2(CCR2) on I/R injury. CCR2 is involved in inducing monocyte chemotaxis to damaged cardiac tissue. Cyp D was related to the progression in early myocardial injury after reperfusion (30-45 min), while CCR2 contributed to I/R injury at a later point after reperfusion (45-60 min). Compared with single deficiency, dual deficiency of Cyp D and CCR2 enhanced cardiac protection regardless of ischemia duration. Moreover, C-reactive protein is commonly used as a marker for an acute inflammatory response and may be associated with myocardial I/R injury directly, due to its inflammatory property [73]. Pei et al. [74] suggested that C-reactive protein directly aggravated I/R injury and that this effect was mainly mediated by the inhibition of mitochondrial ATP-sensitive potassium channels (K-ATP) and the promotion of mPTP opening.

### 2.4. Mitochondrial Calcium Dyshomeostasis

Calcium is believed to exert a vital role in mitochondrial physiology and pathology. Evidence suggests that an upregulation in mitochondrial calcium can boost ATP production by altering the activity of calcium-sensitive mitochondrial matrix enzymes (pyruvate dehydrogenase, isocitrate dehydrogenase, and *α*-ketoglutarate dehydrogenase) [75]. This suggests that the essence of mitochondrial calcium influx is not regulating cytoplasmic calcium, but mitochondrial matrix calcium. In cardiomyocytes, mitochondrial respiration maintains the orderly operation of calcium influx and outflow pathways in mitochondria through electrochemical gradients [76]. The increase of mitochondrial matrix calcium activates the above-mentioned mitochondrial dehydrogenase, increases mitochondrial NADH, promotes electron transfer, and increases ATP production [77]. Mitochondrial calcium transporter (MCU) drives Ca^2+^ into the matrix, and the release of mitochondrial Ca^2+^ is mainly catalyzed by mitochondrial Na^+^/Ca^2+^ exchanger [78, 79]. In addition, mPTP opening may represent a rapid pathway for calcium release [80]. In contrast, severe mitochondrial calcium overload during I/R injury is considered to be a key event that triggers myocytes death [81]. Mitochondrial permeability transition refers to the sudden upregulation in the permeability of inner mitochondrial membrane to solutes with molecular weight up to 1,500 Da, which is due to the mPTP opening [82, 83]. Calcium accumulation through MCU is the main mechanism of calcium-activated mPTP opening, and the inhibition of MCU reduces cardiac I/R injury *in vitro* [84, 85]. The total mitochondrial calcium rather than free matrix calcium might be the trigger for opening the permeability transition [86].

Cardioprotective conditioning strategies are strategies for activating prosurvival pathways and keep cardiac health. Signal transducer and activator of transcription 3 (STAT3) are not only the transcription factors but also exhibit nongenomic prosurvival functions preserving mitochondrial function from I/R injury [87]. It has been reported that the activity of MCU could be influenced by STAT3 [88]. The finding showed that a colocalization/interaction of STAT3 and MCU was observed in rat cardiomyocytes by moderate ROS activation at reperfusion. Phosphorylated STAT3 interacted with MCU, inhibited myocardial I/R injury, and alleviated mitochondrial calcium overload. Phosphorylated STAT3 belongs mainly to the Janus kinase (JAK)/STAT signaling pathway which is a multifunctional pathway that regulates inflammation and cell death [89]. Lemoine et al. [90] proved that atorvastatin administration at early reoxygenation prevented mPTP opening and that was dependent on JAK2/STAT3 signaling pathway in H/R-induced model.

### 2.5. Apoptosis/Necroptosis

The current consensus is that apoptosis is the main form of cardiomyocyte death during myocardial I/R injury [91]. The ultrapermeabilization of the OMM allows the release of mitochondrial death-promoting molecules (Cyt c and second mitochondria-derived activator of caspases) into the cytosol. Then, the former activates caspase-9, which initiates programmed cell death, and the latter reverses the effect of inhibitor of apoptosis proteins and makes it lose the effect of inhibiting caspase activity [92, 93]. This process is also associated with MMP depolarization, ROS overproduction, and B-cell lymphoma 2 (Bcl-2)/Bcl-2 associated X (Bax) downregulation [94–96]. Bax is an effective stimulator of OMM penetration, and Bcl-2 can block the destruction from Bax [97, 98]. The activated Bax will form homooligomers and migrate to the OMM, resulting in enhanced mitochondrial permeability for the escape of apoptotic proteins [99, 100]. One study even revealed that OMM is a key protein that allowed direct activation of Bax [101].

Necroptosis is a regulated pattern of cell death with necrotic appearances, which has been found in various cardiac pathologies, including myocardial I/R injury [102]. The process of mitochondria-mediated necroptosis is not as clear as those of apoptosis. Nevertheless, some key points have emerged. The triggering event of mitochondria-mediated necrosis is the opening of mPTP in inner mitochondrial membrane. The prolonged opening of the mPTP is mediated by core components, for example, Cyp D and VDAC [103, 104]. The mPTP generates nonspecific pores in the inner membrane, causing matrix swelling, mitochondrial respiratory chain dysfunction, and the disorder of ATP production [105–107]. Eventually, ATP exhaustion and extensively disrupted and swollen mitochondrial structure cause cell death through necroptosis. Necroptosis may be regulated by the receptor-interacting serine/threonine-protein kinase 3 (RIPK3). During the reperfusion condition, oxidative stress and calcium overload directly or indirectly activate RIPK3 [108, 109]. In the case of myocardial I/R injury [110, 111], mPTP opening appears to be caused by RIPK3 activation. Calmodulin-dependent protein kinase is a substrate of RIPK3, and activated CaMKII promotes the opening of mPTP. Therefore, RIPK3 expression, CaMKII, and mPTP opening are potential targets for regulating mitochondria-induced necroptosis.

Mitochondria also exert an important role in mediating the cardioprotection of I/R conditioning through a series of signal pathways. Heme oxygenase-1 (HO-1) is a kind of endogenous antioxidants, and HO-1-related signal pathways constitutes an indispensable defense system. Activated by NF-E2-related factor 2 (Nrf2), c-Jun N-terminal kinases (JNK), HO-1, and its metabolites can scavenge hydroxyl-free radicals and superoxide anions and exerts a meaning role in anti-inflammation, antioxidation, and antiapoptosis [112–114]. In response to myocardial H/R injury, HO-1 overexpression preserved the stability of the mitochondrial membrane and reduced mitochondrial ROS overproduction, thereby exerting a protective effect [114]. Silent information regulator protein 1 (SIRT1) is a conserved NAD^+^-dependent histone deacetylase and deacetylates peroxisome proliferator-activated receptor gamma-coactivator 1*α* (PGC1*α*) to promote mitochondrial biogenesis, thereby reducing myocardial cell damage [115, 116]. In addition, SIRT1 deacetylates mitofusin, which contributes to mitochondrial stability under hypoxia [117].

## 3. Cardioprotective Effect of Natural Plant Products in Ischemia/Reperfusion Injury

### 3.1. Polyphenols

Polyphenols are referred to a group of naturally occurring organic compounds named after having multiple phenolic hydroxyl groups [118]. Their advantages include bioactivity, easy availability, specific response, and hypotoxicity [119]. And the public pay close attention to polyphenols because of their ability to oxidative coupling reaction in recent years [120–122]. In addition, rapid metabolism and low bioavailability are their insufficiency [123]. They are generally classified as flavonoids (flavonols, flavanols, flavones, and anthocyanins) and nonflavonoids (phenolic acids, lignans, stilbenes, and tannins) based on the number of phenolic rings and the structural components that bind these rings together in the structure [124, 125]. A wide-ranging medical benefits of polyphenols have now been disclosed, including heart protection [126, 127], anticancer [128], antioxidant [129], anti-inflammatory [130], and antibacterial activities [131]. Polyphenols are reported to play a strong cardioprotective role by regulating oxidative stress response or related signal pathways, especially the antioxidant effect against mitochondrial damage (Table 1).

#### 3.1.1. Cytological Experiments

Chang et al. [132] analyzed the correlation between quercetin-regulated antioxidative stress effect and mitochondria in myocardial H/R model. The results showed that H/R-induced ROS outbreak and MMP depolarization were reversed by quercetin pretreatment. These benefits were related to SIRT1-related pathway mediated by quercetin, which was involved in the regulation of mitophagy and the inhibition of oxidative stress damage. Li et al. [133] investigated the relationship between the activation of K-ATP in cardiomyocytes and Ginkgo biloba extract- (GE-) mediated cardioprotection. Activation of K-ATP by GE reduced H/R-induced damage through the inhibition of mitochondrial fragmentation and MMP depolarization, downregulation of mitochondria-derived O_2_^•−^, and alleviation of mitochondrial calcium overload. Thus, these results suggested that GE exerted a cardioprotective effect and reduced ischemia-caused damage of the functions of heart mitochondria. Min and Wei [134] used H/R injury cell models to investigate the cardioprotection and potential mechanisms of hydroxysafflor yellow A during myocardial I/R injury. The results revealed that it distinctly reduced the level of caspase-3, alleviated ROS injury, and renovated mitochondrial energy metabolism. Further, the mechanism of hydroxysafflor yellow A was liable to depend on the regulation through the PI3K/Akt. Luo et al. [135] explained the targets of ferulic acid for the antioxidant effect on H/R-induced cardiomyocytes. The results showed that ferulic acid inhibited a downward trend of ATP, decreased ROS generation, and maintained MMP. Ferulic acid inhibited mitophagy and reduced the expression of PINK1/Parkin pathway. The findings explained that PINK1/Parkin and mitophagy would be the protective target of ferulic acid. Yu et al. [136] explored the potential mechanisms by which resveratrol reduced cardiomyocyte apoptosis and mitochondrial oxidative damage in H/R model. Resveratrol treatment reduced the excessive generation of ROS in cardiomyocytes and increased the levels of catalase and glutathione peroxidase (GPX). Resveratrol activated PI3K/Akt axis to exert the above effects, which played a direct and positive role in cardiomyocytes during I/R injury.

#### 3.1.2. Animal Experiments

Gonzalez Arbelaez et al. [137] are determined to explore the effects of a polyphenol-enriched cocoa extract (PCE) on I/R injury in normotensive and spontaneously hypertensive rats. The Ii/r model was divided into three groups: nonischemic control hearts, ischemic control hearts, and PCE hearts. They found that PCE reduced infarct size, partly preserved glutathione (GSH), improved the expression of phosphorylated-Akt (p-Akt), phosphorylated glycogen synthase kinase 3 beta (p-GSK-3*β*) and phosphorylated endothelial nitric oxide synthase (p-eNOS), enhanced mPTP response to Ca^2+^, decreased mitochondrial O_2_^•−^ production, inhibited the release of manganese-containing superoxide dismutase and Cyt c, and restored MMP. These findings showed that PCE reduced myocardial I/R injury and suggested that Akt/GSK-3*β*/eNOS pathway was involved. The flavanol (-)-epicatechin (EPI) has attracted much attention as the main active ingredient of cocoa extract. Yamazaki et al. [138] showed solicitude for the cardioprotective effect of EPI after I/R via targeting mitochondrial function. The key to determine the degree of tissue injury is the early events of reperfusion [139]. The results showed that 1 h after reperfusion, EPI treatment demonstrated mitochondria less respiratory inhibition, less mitochondrial loss, lower total mitochondrial calcium, and better-preserved NADH that interrelated to higher ATP level compared with the model group. In addition, EPI stimulated mitochondrial pyruvate transport through NOS/soluble guanylate cyclase pathway, thereby conferring cardioprotection.

Gonzalez Arbelaez et al. [140] established a model of regional ischemia to explore the protection of Ilex paraguariensis extract (IPE) on mitochondrial dysfunction. IPE effectively reduced the infarct size and raised the postischemic myocardial function compared with the control group. The results showed that IPE preserved partially GSH, increased the levels of p-eNOS and p-Akt, and curtailed mPTP opening after IPE administration. These changes were counteracted by a NOS inhibitor. IPE intervention in Akt/eNOS pathway reduced mitochondrial permeability and played a cardioprotective role. Zeng et al. [141] proposed and verified a hypothesis that flavonoids extracted from Dracocephalum moldavica L. (FDM) shielded against myocardial I/R injury via reperfusion injury salvage kinase pathway. Compared with the swelling of mitochondria in the I/R group, the mitochondrial ultrastructure in FDM group remained relatively complete. FDM inhibited the expression of caspase-3, -7, and -9 and increased the ratio of Bcl-2/Bax. Meanwhile, preadministration of FDM promoted the phosphorylation of reperfusion injury salvage kinase pathway. Meng et al. [142] tested a hypothesis that naringenin protected the heart against I/R injury via activating of K-ATP and verified it in the I/R rat model. The results revealed that naringenin restored the cardiac function and narrowed myocardial infarct area. An upregulation in the level of SOD and a downregulation in the activities of MDA in the myocardium were observed after treating naringenin. Interestingly, the cardioprotective effect of naringenin was restrained by 5-hydroxy decanoic acid, a K-ATP blocker. Naringenin could antagonize myocardial I/R injury, which possibly achieved by activating K-ATP on mitochondrial membrane and increasing antioxidative ability of myocardium.

Chiu and Ko [143] studied the time-dependent effects of schisandrin B (Sch B) on GSH and ATP production capacity in the myocardial mitochondria during I/R injury. Sch B administration exerted a time-dependent increasement in mitochondrial GSH, as evidenced by restoring mitochondrial GSH and enhancing the activities of GSH reductase, GPX, and GSH S-transferases, with the reaction achieving maximum at 48 h postdosing and then descending gradually to the control level at 96 h postdosing. The improvement had a bearing on a rising trend in myocardial ATP production ability. It suggested that Sch B could protect the cardiomyocytes against I/R injury by increasing the mitochondrial GSH and ATP production ability, with the optimum cardioprotection demonstrable at 48 h postdosing. Shortly afterwards, the team [144] found that Sch B reduced the susceptibility of mitochondrial Ca^2+^-induced permeability transition, inhibited relevant changes in Ca^2+^-induced MMP, and protected against I/R injury *in vivo*. Liao et al. [145] studied the impacts of long-term (6 weeks) resveratrol preconditioning during I/R injury and its potential mechanisms. The findings showed that resveratrol for long-term intervention prevented mPTP opening and mitochondria-mediated apoptosis, as evidenced by the reduction of Cyt c release, caspase-3 inactivation, and inhibition of apoptotic cells. Furthermore, resveratrol inhibited the I/R-induced increase in VDAC1 expression.

### 3.2. Saponins

Saponins are a variety of compounds generally scattered among the plant kingdom, and their structural characteristics are that they contain a triterpene or steroid aglycone and one or more sugar chains [146]. Such natural products with insufficient biodiversity development may be the important resources for discovery of cost-effective compounds. In addition to the role of plant self-defense, saponins are attached with great importance to pharmaceutical research as active components of a variety of herbs such as potent antineoplastic pharmacophores, antioxidants, neuroprotective agent, or cardioprotective agent [147–150]. Saponins are potential candidates for cardioprotection in clinical of myocardial infarction, but the diversity of these NPPs has not been fully explored, and the specific mechanism remains to be explored. Experimental evidence is summarized in Table 2.

#### 3.2.1. Cytological Experiments

Li et al. [151] studied the antioxidant effect of ginsenoside Rg1 (GsRg1) in H/R-induced model. The group found that GsRg1 administration inhibited cardiomyocyte apoptosis and caspase-3 activity and improved MMP. GsRg1 reduced ROS overproduction by strengthening the antioxidant effect of SOD and decreasing GSH level in cardiomyocytes. In addition, GsRg1 administration might contribute to the nuclear translocation of Nrf2 and increase HO-1 expression, a downstream target gene, in a dose-dependent manner. The results suggested that GsRg1 stimulated the Nrf2/HO-1 pathway to decrease H/R-induced injury. Wang et al. [152] studied the protective effects of ginsenoside Rd (GsRd) on myocardial I/R injury by improving mitochondrial dysfunction. *In vitro* experiment showed that GsRd restrained ROS eruption, inhibited MMP depolarization, and reduced the release of Cyt c from mitochondria to cytoplasm. GsRd inhibited the activation of caspase-9 and -3 and the ratio of Bcl-2/Bax, so as to block apoptotic pathway. A raise of p-Akt and p-GSK-3*β* expressions with GsRd treatment was observed, which suggested that GsRd took effect in I/R injury by blocking the mitochondria-mediated apoptotic pathway via the Akt/GSK-3*β* axis.

#### 3.2.2. Animal Experiments

The SIRT1 signaling pathway could prompt the body's aerobic metabolism and mitochondrial biosynthesis to resist heart damage [153]. Huang et al. [154] found that ginsenoside Rc (GsRc) stimulated SIRT1/PGC1*α* pathway to promote mitochondrial energy metabolism and reduce apoptosis against I/R injury, which provided new evidence for the molecular mechanisms of GsRc as a cardiac protective drug. In the I/R-induced model, GsRc treatment potently promoted mitochondrial ATP production and enhanced the levels of mitochondrial respiratory chain complex II-IV through SIRT1/PGC1*α* pathway. Yu et al. [155] confirmed that gypenosides (GP) could gain benefits during myocardial I/R injury. They demonstrated that GP pretreatment limited infarct size presenting dose dependence in I/R model. GP pretreatment decreased MDA and protected the intracellular antioxidant contents (GPX and SOD). The results also showed that the protective effects of GP were interrelated with the restoration of mitochondrial function of cardiomyocytes, as demonstrated by ATP generation and complex I, II, and IV activities on the mitochondrial respiration chain. Furthermore, GP maintained mitochondrial membrane integrity and inhibited the cytoplasmic translocation of mitochondrial Cyt c. M. Wang et al. [156] revealed that calenduloside E (CE) maintained mitochondrial homeostasis by mediating AMPK pathway and reduced mitochondrial division, thereby effectually attenuating I/R injury. In response to I/R injury, CE played a protective effect by reducing myocardial infarction area and inhibiting apoptosis. For damaged mitochondria, CE restored the ultrastructure, increased ATP content and MMP, and inhibited the prolonged opening of mPTP. The researchers indicated that CE mediated AMPK and intervention with the AMPK inhibitor counteracted the cardioprotective effect of CE on mitochondria.

### 3.3. Terpenoids

Terpenoids are a class of compounds and their derivatives whose molecular skeleton uses isoprene units as basic structural units [157]. Green plants, especially, flowering plants, display an abnormally high amounts of terpenoids, both in species and totality, compared with other organisms [158]. Products downstream of terpenoids such as carotenoids, tanshinone, and paclitaxel demonstrate cardiovascular protective, antioxidant, and anti-inflammatory activities [159–162]. In addition to the above-mentioned effects, the role of terpenoids in attenuating mitochondrial dysfunction cannot be ignored, as illustrated in Table 3.

#### 3.3.1. Cytological Experiments

Lycopene is an effective antioxidant carotenoid that has been proven to have protective effects on IHD. Yue et al. [163] further investigated the capacity for lycopene to protect the myocardial cells operated to H/R and assessed mitochondrial function upon lycopene exposure. Lycopene pretreatment of H/R-induced myocardial cells suppressed the activation of mPTP by decreasing the levels of intracellular ROS and MDA. The depolarization reversal of MMP, an increase of intracellular ATP levels, a downtrend of Cyt c translocated to the cytoplasm, and the inhibition of activated caspase-3 were observed upon receiving lycopene. The protective effect of lycopene could be due to its role on regulating mitochondrial dysfunction in H/R-induced myocardial cells.

#### 3.3.2. Animal Experiments

The microtubule in cardiomyocytes regulates the permeability of OMM through VDAC [164]. Taxol is regarded as a microtubule stabilizer, with the function of stabilizing microtubules [165]. Taxol protected myocardium and mitochondrial function through JNK1/HO-1 pathway during ischemia, which was studied by Cao et al. [166]. The results showed that taxol could effectively reduce ROS outbreak and maintain mitochondrial energy metabolism. After taxol administration, the expression of JNK1 and HO-1 increased, while the JNK inhibitor could inhibit the expression of HO-1. Xiao et al. [167] explored the protective effect of taxol on cardiac functional restoration during reperfusion. The results showed that taxol preserved the intact microtubule structure in reperfusion. There was no change in the expression of mPTP RNA while taxol reduced the mPTP opening, and this impact was confirmed by a decrease in ROS levels. Taxol diminished cell death and the release of mitochondrial Cyt c during I/R injury. It also improved rapid recovery of intracellular calcium concentration, inhibited decrease of the amplitude of Ca^2+^ transients, and shortened the decay time of Ca^2+^transients. The findings revealed that taxol appeared to promote cardiac functional recovery during I/R injury via inhibiting mPTP opening, attenuating anomalous calcium homeostasis.

Li et al. [168] found that tanshinone IIA (Tan IIA) alleviated myocardial I/R injury in rats by mediating PI3K/Akt/rapamycin (mTOR) signaling pathway. Compared with the model group, Tan IIA treatment significantly decreased the expressions of mitochondrial MDA and H2O2 but increased the activities of SOD and SDH. At the same time, Tan IIA promoted the expression of PI3K, Akt, and mTOR. Interestingly, Zhong et al. [169] also put effort into the cardioprotection of Tan IIA underlying cardiac microvascular I/R injury, with a focus on mitochondrial homeostasis. The team found that Tan IIA was a microvascular protective agent and reduced mitochondrial damage and relieved microvascular I/R damage by activating the SIRT1/PGC1*α* pathway. Tan IIA inhibited MMP depolarization and mPTP opening and reduced mitochondrial proapoptotic factor leakage by mediating SIRT1/PGC1*α*, thereby blocking mitochondria-activated programmed death pathway. In contrast, SIRT1 inhibitor dispelled the effective role of Tan IIA on mitochondrial function.

### 3.4. Alkaloids

Alkaloids, broadly defined, are naturally occurring organic nitrogenous compounds which are common in plants, especially in certain families of flowering plants [170]. They have more than 12000 different structures, forming a very large and highly diverse group of secondary compounds [171]. Alkaloids have been used as antipyretic, sedative, and detoxifying drugs for thousands of years [172]. In modern medicine, alkaloids involve in a multiple pharmacological functions, such as analgesic, antiarrhythmic, anticancer, antibacterial, and antihyperglycemic effects [173–176]. However, studies on the weakening effects of alkaloids in myocardial I/R injury primarily focus on anticardiomyocyte apoptosis, and a consensus on the effects of alkaloids on improving mitochondrial dysfunction has not been reached. The relevant experimental evidence is summarized in Table 4.

#### 3.4.1. Cytological Experiments

Bai et al. [177] explored the role of K-ATP in protective effects of anisodamine on I/R injury. The results showed that anisodamine remarkably improved the hemodynamic indexes in I/R-induced model, reduced the infracted myocardial area, and improved the myocardial and mitochondrial ultrastructural damages. The *in vitro* results confirmed that anisodamine improved mitochondrial energy metabolism, downregulated MDA and SOD, and stabilized MMP. The protective effects were eliminated by a K-ATP blocker, which revealed that the opening of K-ATP exerted a decisive role in the cardioprotective role of anisodamine against myocardial I/R injury. Zhang et al. [178] confirmed that tetrandrine played a protective effect during I/R injury, as evidenced by effectively reducing the level of caspase-3, restoring mitochondrial energy metabolism in myocardial cells. Then, it was further found that tetrandrine played the above role through JAK3/STAT3 pathway. Pathway inhibitor tests supported this finding.

#### 3.4.2. Animal Experiments

Wang et al. [179] explored the protective effects of berberine on I/R injury and its effect on mitochondrial dysfunction. Rats were intervened with berberine for 4 weeks, and then, I/R was executed. The results suggested that berberine decreased myocardial infarction area compared with I/R group. Berberine improved mitochondrial dysfunction via MMP and complex I. Berberine heightened the expression of Bcl-2 and mitochondrial Cyt c and inhibited the expression of Bax and cytosolic Cyt c. Overall, berberine protected myocardial cells from I/R through alleviating mitochondrial dysfunction and myocardial apoptosis. Lee et al. [180] demonstrated that HO-1 exerted a vital role for the cardioprotective effect of higenamine against I/R-related damage. The damage was associated with mitochondria-mediated apoptosis as underlined by a rise of Cyt c release and caspase-3. The results revealed that an upregulation in the level of Bcl-2 and HO-1 and a downregulation in the expression of Bax, cytosolic Cyt c, and caspase-3 receiving higenamine pretreatment were observed. Most importantly, administration of an enzyme inhibitor of HO-1 suppressed the positive role of higenamine. Thus, NPPs to stimulate HO-1 tends to be a reasonable therapeutic target to decrease the threat of I/R-related injury.

## 4. Conclusion

**Figure 2:**
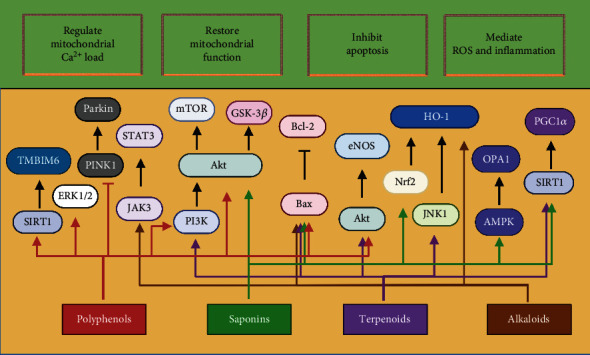
Cardioprotective effects of natural plant products targeting mitochondria in myocardial ischemia/reperfusion injury. TMBIM6: transmembrane BAX inhibitor-1 motif-containing 6; SIRT1: silent information regulator protein 1; eNOS: endothelial nitric oxide synthase; GSK: glycogen synthase kinase; ERK: extracellular signal-regulated kinase; STAT3: signal transducer and activator of transcription 3; JAK3: Janus kinase 3; mTOR: mammalian target of rapamycin; PGC1*α*: peroxisome proliferator-activated receptor gamma-coactivator 1*α*; HO-1: heme oxygenase 1; Nrf2: NF-E2-related factor 2; PINK1: phosphatase and tensin homolog-induced putative kinase 1; JNK1: c-Jun N-terminal kinases 1; AMPK: AMP-activated protein kinase; OPA1: optic atrophy 1. Created with http://BioRender.com.

NPPs always play the part of new clue for pharmaceutical development in the past. The review focuses on NPPs that have been identified in the recent years with the therapeutic effect targeting mitochondria and their origin and structural classification. These NPPs mainly improve mitochondrial dysfunction and inhibit apoptosis, as shown in Figure 2. However, the current use of NPPs is still facing considerable challenges. Because of the complexity of molecular mixtures, it is often difficult to find new drug candidates from natural products. The therapeutic activity of natural plant extracts is usually due to the synergistic and simultaneous action of several chemical substances. The good news is that emerging new technologies provide new solutions. Through quantum computing, microfluidics, molecular docking, and other technologies, researchers use combinatorial methods to take advantage of the therapeutic properties of NPPs while studying their molecular effects under physiological conditions [181–184]. Among them, molecular docking has become an important tool of the drug discovery process [185]. Over the years, the modalities have moved from being a stand-alone approach to being employed in combination with other computational approaches within integrated workflows, which means the generation of an “ensemble” of drug target conformations in computational structure-based drug discovery, often obtained by using molecular dynamics simulation, that is used in docking candidate ligands [186]. Zuo et al. [187] used molecular docking to prove that isovaleroylbinankadsurin A protected the myocardial cells from I/R injury through activating glucocorticoid receptor and consequently inhibiting the ROS generation. Molecular docking is also in favor of the extended search for mitochondria-targeted drugs. Liu et al. [188] first demonstrated that paeonol is a novel mitochondrial fusion promoter through molecular docking, which protected against hyperglycemia-induced mitochondrial oxidative injury in the myocardial cells. In recent years, the understanding of mitochondrial dysfunction in I/R injury has increased at express speed, but the researches on NPPs targeted drugs are still in the early stage. Future research still needs to translate new discoveries into potential treatments.

## Figures and Tables

**Table 1:** Detected studies reporting cardioprotective effects of polyphenols against mitochondrial damage in I/R injury.

Author	NPPs	Source	Experiment	Model	Administration	Dosage	Target	Ref.
Xing Chang	Quercetin	Ginkgo biloba	H/R	Human cardiomyocytes	Incubate	50, 100, 150, 200, and 250 mg/L	ROS, MMP, SIRT1/TMBIM6, and MRF	[132]
Tonghua Li	EGb761	Ginkgo biloba	H/R	H9c2 cells	Incubate	100 mg/ml, 2 *μ*mol/L	MitoBK_Ca_, superoxide	[133]
Jia Min	Hydroxysafflor yellow A	Carthamus tinctorius L.	H/R	H9c2 cells	Incubate	1.25, 5, and 20 *μ*mol/L	MEM, PI3K/Akt/HKII	[134]
Chenxi Luo	Ferulic acid	Cimicifuga foetida L.	H/R	H9c2 cells	Incubate	12.5 *μ*mol/L	MPP, ROS, and PINK1/Parkin	[135]
Dongmin Yu	Resveratrol	Grape	H/R	NPCMs	Incubate	1, 2, 4 *μ*mol/L	PI3K/Akt, ROS	[136]
Luisa F González Arbelaez	Flavan-3-ols, (-)-epicatechin, and procyanidin B2	Cocoa	I/R	SHR; Wistar rats	Perfuse	30 *μ*g/mL	Ca^2+^-induced mPTP opening, MMP, and Akt/GSK-3*β*/eNOS	[137]
Katrina Go Yamazaki	(-)-Epicatechin	Cocoa	I/R	SD rats	Intravenous administration	10, 20 mg/kg	MRR, NADH, MMS, and NOS/sGC	[138]
Luisa F González Arbelaez	Rutin, caffeine, and chlorogenic acid	Ilex paraguariensis	I/R	Wistar rats	Perfuse	30 *μ*g/mL	mPTP opening, Akt/eNOS, and GSH	[140]
Cheng Zeng	Luteolin-7-O-*β*-D-glucuronide, apigenin-7-O-*β*-D-glucuronide, diosmetin-7-O-*β*-D-glucuronide	Dracocephalum Moldavica L.	I/R	SD rats	Gavage	3, 10, 30 mg/kg	MMS, PI3K/Akt/GSK-3*β*, and ERK1/2	[141]
Limin Meng	Naringenin	Citrus	I/R	SD rats	Perfuse	1.25, 2.5, 5, 10, 20, and 40 *μ*mol/L	ROS, K-ATP	[142]
Po Yee Chiu	Schisandrin B	Fructus Schisandrae	I/R	SD rats	Gavage	1.2 mmol/kg	GSH, MEM	[143]
Po Yee Chiu	Schisandrin B	Fructus Schisandrae	I/R	SD rats	Gavage	1-2 mmol/kg	Ca^2+^-induced mPT, and ROS	[144]
Zhangping Liao	Resveratrol	Grape	I/R	Kun-Ming mice	Gavage	2 mg/kg	VDAC1, Cyt c, and mPTP opening	[145]

NPPs: natural plant products; I/R: ischemia-reperfusion; SHR: spontaneously hypertensive rats; mPTP: mitochondrial permeability transition pore; MMP: mitochondrial membrane potential; TMBIM6: transmembrane BAX inhibitor-1 motif-containing 6; SIRT1: silent information regulator protein 1; NPCMs: neonatal rat primary cardiomyocytes; MRR: mitochondrial respiration rate; NOS: nitric oxide synthase; sGC: soluble guanylate cyclase; GSK: glycogen synthase kinase; ROS: reactive oxygen species; PI3K: phosphatidylinositol-3-kinase; ERK: extracellular signal-regulated kinase; NADH: nicotinamide adenine dinucleotide; NADPH: nicotinamide adenine dinucleotide phosphate; HR: hypoxia/reoxygenation; MitoBK_Ca_: mitochondrial large-conductance Ca^2+^-activated K^+^ channels; GSH: glutathione; MRF: mitochondrial respiratory function; SD: Sprague-Dawley; MMS: myocardial mitochondrial structure; MEM: mitochondrial energy metabolism; K-ATP: adenosine triphosphate-sensitive potassium channel; ERK: extracellular signal-regulated kinase: Cyt c, cytochrome c; PINK1: phosphatase and tensin homolog-induced putative kinase 1; VDAC1: voltage-dependent anion channel 1.

**Table 2:** Detected studies reporting cardioprotective effects of saponins against mitochondrial damage in I/R injury.

Author	NPPs	Source	Experiment	Model	Administration	Dosage	Target	Ref.
Qianhui Li	Ginsenoside Rg1	Panax ginseng	H/R	H9c2 cells	Incubate	10, 20, 40, and 60 *μ*mol/L	MMP, ROS, and Nrf2/HO-1	[151]
Yang Wang	Ginsenoside Rd	Panax ginseng	I/R; H/R	SD rats; NPCMs	Intravenous administration; incubate	50 mL/kg; 10 *μ*mol/L	MMP, Akt/GSK-3*β*, and Cyt c	[152]
Qingxia Huang	Ginsenoside Rc	Panax ginseng	I/R; H/R	Kunming mice; H9c2 cells	Gavage; incubate	10 mL/kg; 2.5, 5, and 10 *μ*mol/L	ATP, SIRT1/PGC1*α*	[154]
Haijie Yu	Gypenosides	Gynostemma pentaphyllum	I/R	Wistar rats	Gavage	50, 100, and 200 mL/kg	ROS, ATP, Cyt c, and MRF	[155]
Min Wang	Calenduloside E	Aralia elata (Miq.) Seem	I/R	SD rats	Gavage	15 mg/kg	MMP, mPTP opening, and AMPK/OPA1	[156]

HO-1: heme oxygenase 1; Nrf2: NF-E2-related factor 2; AMPK: AMP-activated protein kinase; OPA1: optic atrophy 1.

**Table 3:** Detected studies reporting cardioprotective effects of terpenoids against mitochondrial damage in I/R injury.

Author	NPPs	Source	Experiment	Model	Administration	Dosage	Target	Ref.
Rongchuan Yue	Lycopene	Tomato	H/R	NPCMs	Incubate	5 *μ*mol/L	ROS, mPTP, Cyt c, and caspase-3	[163]
Huaming Cao	Taxol	Taxus	I/R	SD rats	Perfuse	0.1, 0.3, and 1 *μ*g/mL	ROS, JNK1/HO-1	[166]
Junjie Xiao	Taxol	Taxus	I/R	SD rats	Perfuse	0.1, 0.3, 1 *μ*g/mL	ROS, mPTP opening, and Cyt c, Ca^2+^	[167]
Qiang Li	Tanshinone IIA	Salvia miltiorrhiza Bunge	I/R; H/R	SD rats; NPCMs	Intravenous administration; incubate	10, 20 mg/kg; 1, 10 *μ*mol/L	ROS, PI3K/Akt/mTOR	[168]
Jiankai Zhong	Tanshinone IIA	Salvia miltiorrhiza Bunge	I/R; H/R	C57BL/6 mice; CMECs	—	5, 25 mg/kg; —	MMP, mPTP opening, and SIRT1/PGC1*α*	[169]

mTOR: mammalian target of rapamycin; CMECs: cardiac microvascular endothelial cells; PGC1*α*: peroxisome proliferator-activated receptor gamma-coactivator 1*α*; JNK1: c-Jun N-terminal kinases 1.

**Table 4:** Detected studies reporting cardioprotective effects of alkaloids against mitochondrial damage in I/R injury.

Author	NPPs	Source	Experiment	Model	Administration	Dosage	Target	Ref.
Shiru Bai	Anisodamine	Anisodus	I/R; H/R	SD rats; NPCMs	Perfuse; incubate	0.3 mmol/L; 10^−6^ mmol/L	K-ATP, MMP, MEM, and MMS	[177]
Tiejun Zhang	Tetrandrine	Stephania tetrandra S Moore	H/R	H9c2 cells	Incubate	1, 5, 25 mmol/L	MMP, JAK 3/STAT 3/HK II	[178]
Yongjun Wang	Berberine	Coptis chinensis	I/R	SD rats	Gavage	200 mg/kg	MMP, Cyt c	[179]
Young Soo Lee	Higenamine	Aconite	I/R	SD rats	Intraperitoneal administration	1, 5, and 10 mg/kg	Cyt c, HO-1	[180]

STAT3: signal transducer and activator of transcription 3; JAK3: Janus kinase 3; HKII: hexokinase II.
